# Inhibition of Arenaviruses by Combinations of Orally Available Approved Drugs

**DOI:** 10.1128/AAC.01146-20

**Published:** 2021-03-18

**Authors:** Shawn Herring, Jessica M. Oda, Jessica Wagoner, Delaney Kirchmeier, Aidan O’Connor, Elizabeth A. Nelson, Qinfeng Huang, Yuying Liang, Lisa Evans DeWald, Lisa M. Johansen, Pamela J. Glass, Gene G. Olinger, Aleksandr Ianevski, Tero Aittokallio, Mary F. Paine, Susan L. Fink, Judith M. White, Stephen J. Polyak

**Affiliations:** a Department of Laboratory Medicine and Pathology, University of Washington, Seattle, Washington, USA; b Department of Cell Biology, University of Virginia, Charlottesville, Virginia, USA; c Department of Veterinary and Biomedical Sciences, University of Minnesota, Twin Cities, Minnesota, USA; d Virology Division, United States Army Medical Research Institute of Infectious Diseases, Frederick, Maryland, USA; e Horizon Discovery, Cambridge, Massachusetts, USA; f MRIGlobal, Gaithersburg, Maryland, USA; g Institute for Molecular Medicine Finland (FIMM), University of Helsinki, Helsinki, Finland; h Oslo Centre for Biostatistics and Epidemiology (OCBE), University of Oslo, Oslo, Norway; i Institute for Cancer Research, Oslo University Hospital, Oslo, Norway; j Department of Pharmaceutical Sciences, Washington State University, Spokane, Washington, USA; k Department of Microbiology, University of Virginia, Charlottesville, Virginia, USA; l Department of Global Health, University of Washington, Seattle, Washington, USA; m Department of Microbiology, University of Washington, Seattle, Washington, USA

**Keywords:** arenavirus, filovirus, Lassa, Junin, Pichinde, Ebola, Marburg, arbidol, repurposing, broad-spectrum antiviral, SARS-CoV-2, synergy, SynergyFinder2

## Abstract

Neglected diseases caused by arenaviruses such as Lassa virus (LASV) and filoviruses like Ebola virus (EBOV) primarily afflict resource-limited countries, where antiviral drug development is often minimal. Previous studies have shown that many approved drugs developed for other clinical indications inhibit EBOV and LASV and that combinations of these drugs provide synergistic suppression of EBOV, often by blocking discrete steps in virus entry. We hypothesize that repurposing of combinations of orally administered approved drugs provides effective suppression of arenaviruses. In this report, we demonstrate that arbidol, an approved influenza antiviral previously shown to inhibit EBOV, LASV, and many other viruses, inhibits murine leukemia virus (MLV) reporter viruses pseudotyped with the fusion glycoproteins (GPs) of other arenaviruses (Junin virus [JUNV], lymphocytic choriomeningitis virus [LCMV], and Pichinde virus [PICV]). Arbidol and other approved drugs, including aripiprazole, amodiaquine, sertraline, and niclosamide, also inhibit infection of cells by infectious PICV, and arbidol, sertraline, and niclosamide inhibit infectious LASV. Combining arbidol with aripiprazole or sertraline results in the synergistic suppression of LASV and JUNV GP-bearing pseudoviruses. This proof-of-concept study shows that arenavirus infection *in vitro* can be synergistically inhibited by combinations of approved drugs. This approach may lead to a proactive strategy with which to prepare for and control known and new arenavirus outbreaks.

## Abstract (precis)

Neglected diseases caused by arenaviruses such as Lassa virus (LASV) and filoviruses like Ebola virus (EBOV) primarily afflict resource-limited countries, where antiviral drug development is often minimal. Previous studies have shown that many approved drugs developed for other clinical indications inhibit EBOV and LASV and that combinations of these drugs provide synergistic suppression of EBOV, often by blocking discrete steps in virus entry.

## INTRODUCTION

Members of the *Arenaviridae*, a family of enveloped viruses with a segmented RNA genome, are capable of causing hemorrhagic fevers and neurological diseases (1–3). Among its three genera, mammarenaviruses are known to cause disease in humans. Mammarenaviruses are further classified into Old World (OW) and New World (NW) arenaviruses, with outbreaks occurring in Africa and the Americas, respectively. OW arenaviruses include Lassa virus (LASV) and lymphocytic choriomeningitis virus (LCMV). LASV is estimated to cause ∼100,000 to 300,000 human infections and ∼5,000 human deaths per year. LCMV, which is deleterious in pregnant women and transplant recipients (4), is present globally. NW arenaviruses include Junin (JUNV), Pichinde (PICV), Chapare (CHAPV), and Tacaribe (TACV) viruses. JUNV has caused outbreaks of hemorrhagic fever in Argentina, with an estimated case fatality rate of 15 to 30%. Although an effective vaccine has been developed (5), JUNV infections are generally treated with supportive care and in some cases convalescent-phase serum. Aside from these countermeasures and the use of ribavirin for severe cases of LASV (6), there are no approved vaccines, therapeutic antibodies, or drugs with which to treat patients infected with an arenavirus.

Drug screens have identified small molecules with activity against LASV and other arenaviruses. Most are investigational drugs and include clotrimazole derivatives (7), ST-193 (8, 9), F3406 (10), LHF-535 (11), kinase inhibitors (12, 13), losmapimod (14), and inhibitors of purine and pyrimidine biosynthesis (15–17). The polymerase inhibitors remdesivir and favipiravir have shown some activity against arenaviruses in cell cultures (18) and *in vivo* (19), respectively. Approved drugs that surfaced in arenavirus drug screens were mycophenolic acid, a broad-spectrum inhibitor of purine biosynthesis (20); leflunomide, an inhibitor of pyrimidine biosynthesis (17); the calcium channel blockers lacidipine (21), nifedipine, and verapamil; and gabapentin (22). Several of these drugs (e.g., ST-193 and F3406) block the entry stage, while others (e.g., remdesivir and the purine and pyrimidine synthesis inhibitors) block the replication stage of the arenavirus life cycle.

We are interested in identifying synergistic combinations of approved drugs for use at the inception of new viral outbreaks. The concept is that once the family of the causative virus is identified by genomic sequencing, for example, a filovirus, an arenavirus, or a coronavirus, there would be a shelf-ready cocktail of approved drugs for immediate use. A cocktail documented in advance to reduce titers by multiple members of the implicated virus family would be highly beneficial. Approved drugs have many positive features for this purpose, including shelf-ready availability, relatively low cost, room-temperature stability, delivery by the oral route, utility in nonhospitalized settings, and known pharmacology (23, 24). We favor an approach employing combinations of approved drugs, as a frequent limitation of monotherapy with a drug approved for another indication is the inability to achieve viral suppression (reflected in the concentration of the drug that suppresses a virus by 50% [50% inhibitory concentration {IC_50_}]) at concentrations that are clinically achievable. With synergistic drug combinations, the dose of the individual drugs needed for antiviral activity is lowered, thereby allowing the maximum serum concentration (*C*_max_) to approach, equal, or exceed the IC_50_ while reducing the risk of adverse effects. Other advantages of drug combination approaches are possible reductions in the development of viral resistance (25).

Toward the goal of developing combinations of approved drugs against (re)emerging viral infections, we (26) and others (27) identified combinations of approved drugs that synergistically suppress Ebola virus (EBOV) in cell cultures. The emphasis in both studies was on drugs that block virus entry, the first step in all viral life cycles. To begin to broaden this approach, we further identified approved drugs with activity against both EBOV (a filovirus) and LASV (an OW arenavirus) (28). While EBOV and LASV utilize different cell surface attachment factors and endosomal receptors, they share important features in their fusion and entry mechanisms: (i) both viruses display multiple copies of a trimeric class I fusion glycoprotein termed GP; (ii) each trimer is composed of heterodimers combining a receptor binding subunit and a fusion subunit, with the latter containing an internal fusion loop; (iii) the viruses are taken into cells by macropinocytosis; and (iv) the viral GPs are triggered by a combination of binding to an endosomal receptor and exposure to low endosomal pH, which converts the GPs to their fusogenic, trimer-of-hairpins conformation (reviewed in references 29 and 30).

Reflecting these shared traits and features of virus entry, several approved drugs suppress the entry of both EBOV and LASV into cells. In the current study, we pursued combination testing of arbidol (Arb), a fusion blocker, with several other approved drugs against LASV and JUNV GP-bearing pseudoviruses (PVs). Arbidol, which inhibits influenza virus entry by inactivating the viral hemagglutinin (HA) protein (31), also inhibits hepatitis C virus, EBOV, LASV, JUNV, TACV, Zika virus, and severe acute respiratory syndrome coronavirus 2 (SARS-CoV-2), perhaps through a similar mechanism (28, 32–39). We also evaluated drugs previously shown to inhibit EBOV and/or LASV at discrete steps of the virus entry pathway: aripiprazole (Ari) (an antipsychotic drug that blocks macropinocytotic internalization) (26, 28), amodiaquine (Amo) (a lysosomotropic antimalarial drug that increases endosome pH) (26, 28, 40), sertraline (Sert) (an antidepressant that blocks fusion) (23, 26, 28, 41), and niclosamide (an anthelminthic drug that inhibits the entry of several viruses) (42–46) (see Fig. S1 in the supplemental material). We demonstrate that in addition to blocking EBOV, LASV, and TACV (28, 32), arbidol blocks entry mediated by the GPs of other arenaviruses, including LCMV, JUNV, and PICV, and filoviruses such as Marburg virus (MARV). Moreover, arbidol, amodiaquine, aripiprazole, sertraline, and niclosamide also inhibit infection of cells by infectious PICV, and arbidol, sertraline, and niclosamide also inhibit fully infectious LASV. We further show that the combinations of arbidol plus aripiprazole and arbidol plus sertraline synergistically suppress infections mediated by the GPs of LASV and JUNV.

## RESULTS

### Arbidol inhibits multiple arenaviruses.

**FIG 1:**
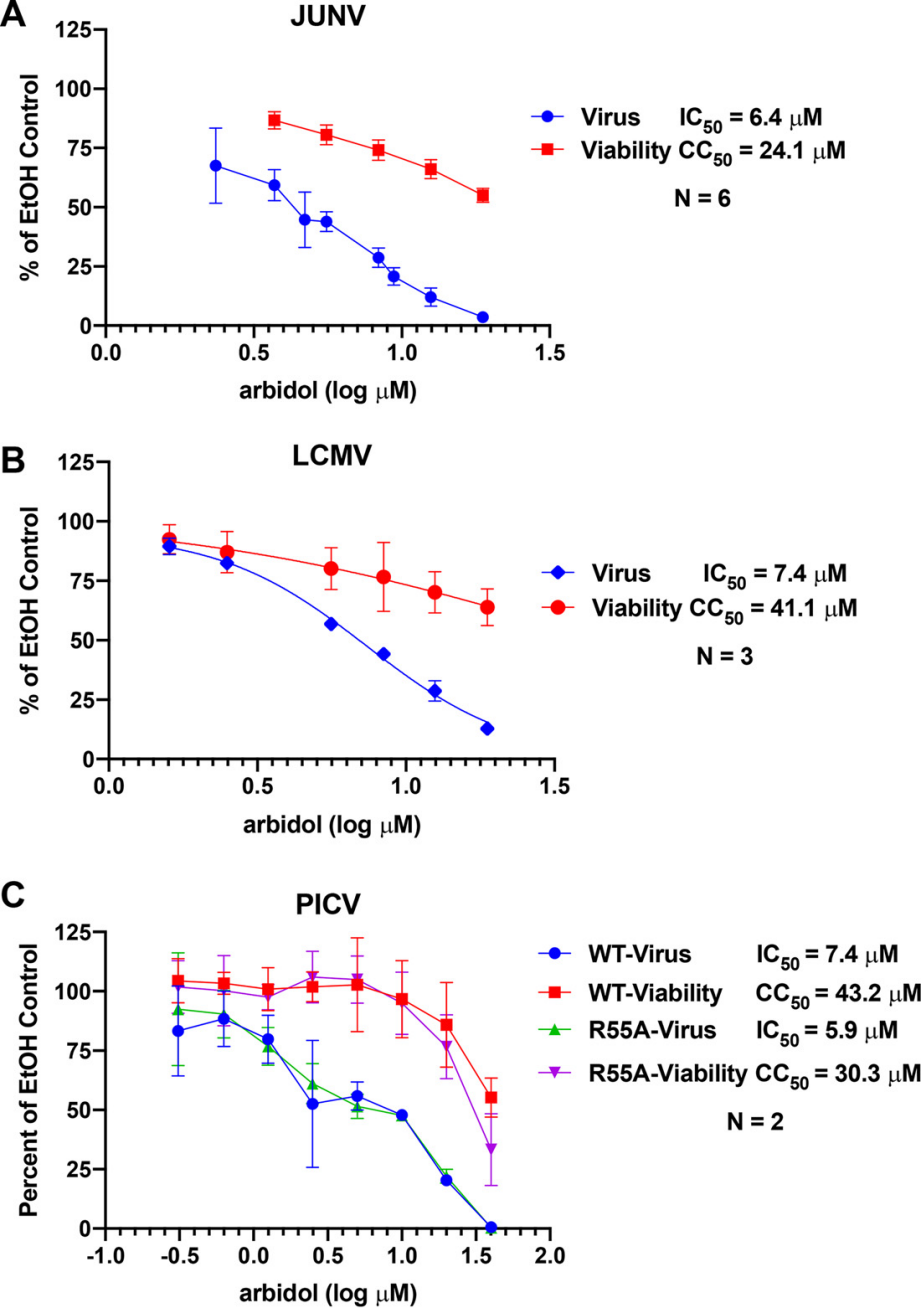
Arbidol inhibits pseudoviruses expressing multiple arenavirus glycoproteins. Vero cells were treated with various concentrations of arbidol prior to infection with MLV pseudoviruses that enter cells via the glycoprotein of JUNV, LCMV, PICV WT, or PICV R55A. Twenty-four hours later, luciferase activity was measured to quantify virus infection, and ATP levels were measured to quantify cell viability. Error bars represent standard deviations. Each condition was performed in triplicate, and each experiment was performed 6, 3, 2, and 2 times for JUNV, LCMV, PICV WT, and PICV R55A, respectively. For each virus, the data depict the averages and standard deviations across all experiments performed for that virus. IC_50_ and CC_50_ values were calculated in Prism. EtOH, ethanol.

As our previous studies revealed that arbidol inhibits infection mediated by the LASV and EBOV GPs (28, 32), we first tested whether arbidol inhibits infection mediated by the GPs of other arenaviruses and filoviruses. Murine leukemia virus (MLV) luciferase reporter viruses pseudotyped with LASV, JUNV, LCMV, EBOV, MARV Angola, and MARV Musoke GPs were generated, and the expression of the relevant GP was confirmed in pseudovirus stocks (see Fig. S2 in the supplemental material). Arbidol inhibited infection of Vero cells with MLV reporter viruses pseudotyped with GPs from the arenaviruses JUNV, LCMV, and PICV (Fig. 1). For PICV, we tested pseudoviruses carrying wild-type (WT) PICV GP and a mutant (R55A) PICV GP, which was previously shown to have enhanced fusion activity (47). Arbidol inhibited infection of cells with all four pseudoviruses, with IC_50_s of 6.4, 7.4, 7.4, and 5.9 μM for JUNV, LCMV, PICV WT, and PICV R55A, respectively (Fig. 1). The selectivity indices (50% cytotoxic concentration [CC_50_]/IC_50_) were 3.8, 5.6, 5.8, and 5.1, respectively. Arbidol also inhibited MLV pseudoviruses bearing filovirus GPs from MARV Angola and MARV Musoke, with IC_50_s of 3.9 and 3.5 μM and selectivity indices of 13.2 and 10.6, respectively (Fig. S3).

### Approved drugs inhibit infectious PICV and LASV.

**FIG 2:**
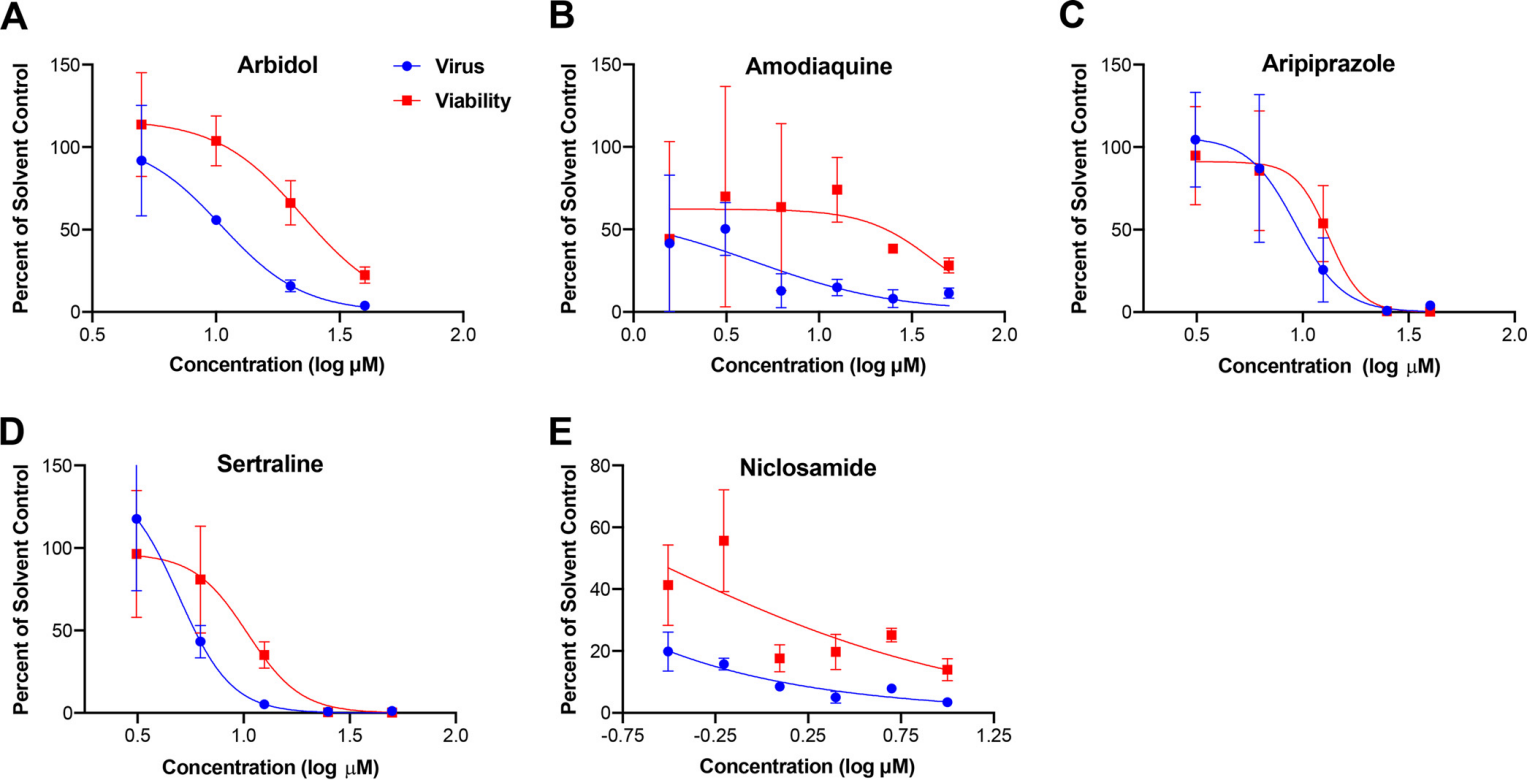
Arbidol and other approved drugs inhibit infectious PICV. Vero E6 cells were treated for 1 h with the indicated concentrations of arbidol (A), amodiaquine (B), aripiprazole (C), sertraline (D), and niclosamide (E) and infected with PICV-GFP at an MOI of 0.1. Forty-eight hours later, cells were fixed, and nuclei were counterstained with DAPI. All conditions were conducted in triplicate, and experiments were repeated 4, 1, 2, 3, and 3 times for arbidol, amodiaquine, aripiprazole, sertraline, and niclosamide, respectively. A representative curve is shown for each drug. Virus infection and cell viability were quantified by measuring GFP and DAPI fluorescence using a Cytation 1 imaging platform. For amodiaquine, cell viability was assessed by an ATPlite assay.

We extended studies of arbidol and the approved drugs amodiaquine, aripiprazole, sertraline, and niclosamide versus arenaviruses using biosafety level 2 (BSL2)-compatible infectious PICV containing a green fluorescent protein (GFP) reporter (48). The PICV-GFP virus is a fully replication-competent arenavirus that encodes all WT viral proteins and RNA elements and utilizes the same mechanism of replication as WT PICV, including PICV GP-mediated cell entry, L- and NP-dependent viral RNA replication and transcription, and matrix protein Z-mediated virus assembly and budding (48). All five drugs inhibited PICV-GFP infection of Vero E6 cells with IC_50_s of 8.4, 4.5, 5.4, 3.7, and <0.2 μM for arbidol, amodiaquine, aripiprazole, sertraline, and niclosamide, respectively (Fig. 2; Table S1). Moreover, arbidol, sertraline, and niclosamide inhibited infectious LASV with similar IC_50_s of 10, 7, and 0.2 μM, respectively (Table S1). Collectively, the data indicate that arbidol, amodiaquine, aripiprazole, sertraline, and niclosamide inhibit multiple arenaviruses.

### Arbidol synergizes with other approved drugs to suppress arenavirus GP-mediated infection.

**FIG 3:**
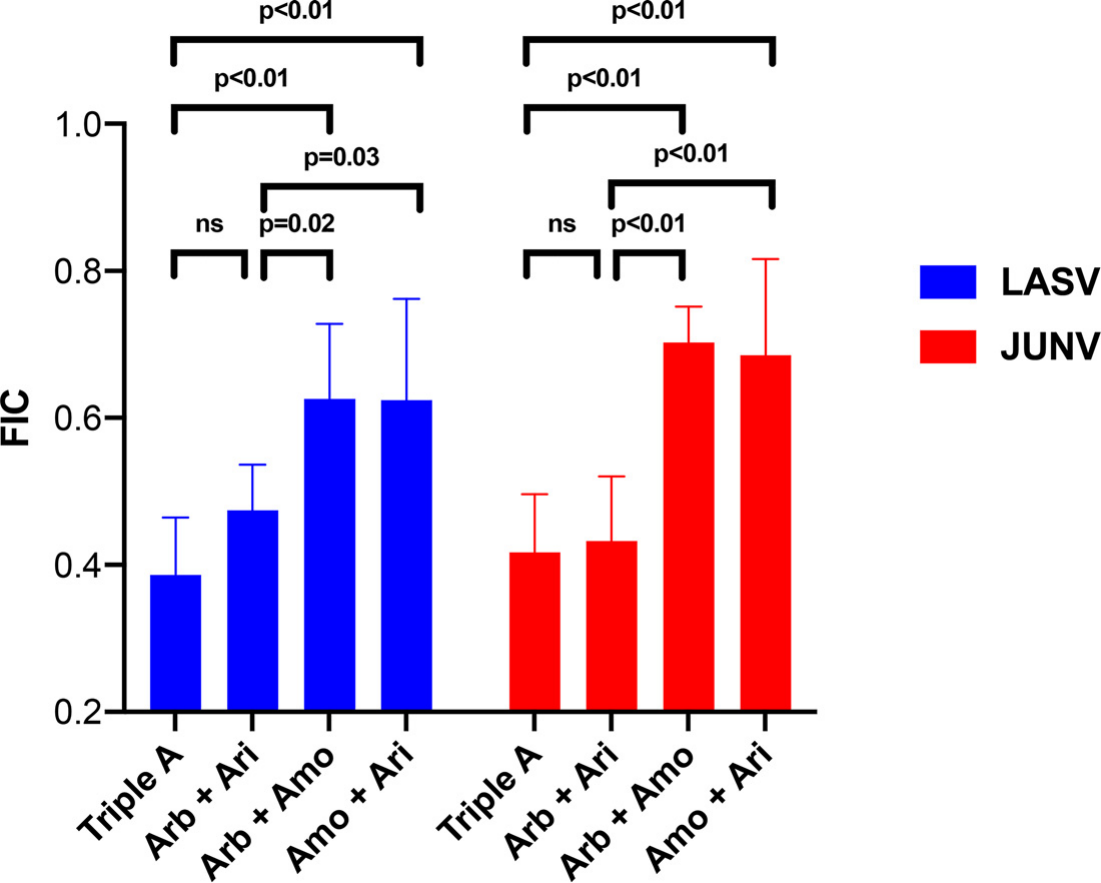
Arbidol combined with the approved drug aripiprazole causes synergistic suppression of LASV and JUNV glycoprotein-bearing pseudoviruses. Vero cells were treated with various concentrations of a single drug or a two- or three-drug combination of arbidol (Arb), amodiaquine (Amo), and aripiprazole (Ari) before infection with LASV or JUNV pseudovirus. Triple A refers to the combination of the three drugs. At 24 h postinfection, luciferase activity was measured. To quantify the combination effects for each experiment, the fractional inhibitory concentration (FIC) score was calculated by dividing the observed IC_50_ by the expected IC_50_ for each combination. For LASV, data represent averages and standard deviations from 8 separate experiments, with Z scores of >0.6 (the average Z score was 0.71 ± 0.06) and where each condition was conducted in triplicate. The 1× concentrations of Arb, Amo, and Ari were 11, 8, and 12 μM, respectively. For JUNV, data represent averages and standard deviations from 6 separate experiments, with Z scores of >0.2 (the average Z score was 0.5 ± 0.11) and where each condition was conducted in triplicate. The 1× concentrations of Arb, Amo, and Ari were 8, 12, and 5 μM, respectively. FICs of <1 suggest synergy. *P* values are derived from one-way ANOVA using Tukey’s multiple-comparison test in GraphPad Prism. ns, not significant.

We first evaluated combinations of arbidol (fusion inhibitor), amodiaquine (endosomal acidification inhibitor), and aripiprazole (virus internalization inhibitor) using drug combination assay 1 (see Materials and Methods and Fig. S4 in the supplemental material). Figure 3 presents average fractional inhibitory concentration (FIC) data for the two- and three-drug combinations against LASV-GP and JUNV-GP pseudovirus infections of Vero cells. The FIC is a ratio of the observed IC_50_ to the expected IC_50_ for each two-drug and three-drug combination. The expected IC_50_ for the two- or three-drug combinations is the arithmetic mean of the IC_50_ for each drug tested individually. By examining the single-drug dose-response data, the concentration closest to 50% inhibition was designated the observed IC_50_. FICs of 1 suggest additivity, FICs of >1 suggest antagonism, and FICs of <1 suggest synergism (49, 50). All drug combinations yielded FICs of <1, suggesting synergy. Figure S5 depicts the antiviral concentration-response curves for the single-, double-, and triple-drug combinations. The triple-drug combination of arbidol plus amodiaquine and aripiprazole (referred to as “triple A”) and the two-drug combination of arbidol plus aripiprazole produced the lowest and similar FICs for suppression of LASV and JUNV infection (Fig. 3). Moreover, the triple-A combination and the two-drug combination of arbidol plus aripiprazole produced FICs that were statistically significantly lower (suggesting the greatest synergy) than those for the other two-drug combinations arbidol plus amodiaquine and amodiaquine plus aripiprazole (Fig. 3). When viewed from the perspective of the plate map, two- and three-drug combinations required low concentrations to achieve approximate IC_50_ suppression of virus infection compared to the single drugs, and drug combinations yielded FICs of <1, suggesting synergy (Table 1). Thus, the two- and three-drug combinations lowered the IC_50_ for inhibition of LASV and JUNV pseudoviruses. For example, using the formula for percent reduction {[(monotherapy concentration − combination concentration)/monotherapy concentration] × 100}, the triple-A combination and the two-drug combination of arbidol plus aripiprazole yielded approximately 70% reductions in the concentrations of arbidol and aripiprazole needed for LASV and JUNV suppression.

**TABLE 1:**
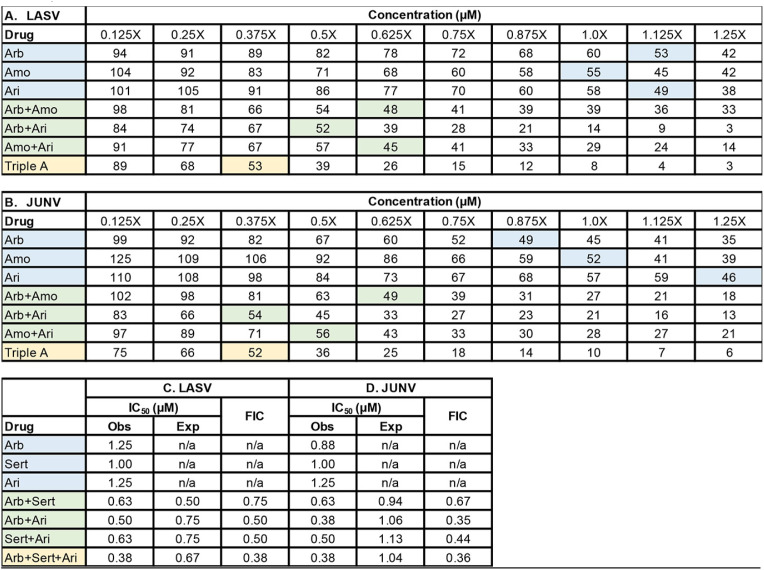
Representative plate maps of synergistic combination testing against LASV and JUNV pseudoviruses^*a*^

a Vero cells were treated with the indicated concentrations of a single drug or a two- or three-drug combination of arbidol (Arb), amodiaquine (Amo), and aripiprazole (Ari) before infection with LASV (A) or JUNV (B) pseudovirus. Triple A refers to the combination of the three drugs. Data are expressed as a percentage of infected cells treated with the solvent control. At 24 h postinfection, luciferase activity was measured. The concentration of each drug alone providing ∼50% inhibition of infection is shaded blue. The concentration of each drug needed in the pairwise combinations to produce ∼50% inhibition of infection is shaded green. The concentration of each drug needed in the three-drug cocktail to yield ∼50% inhibition of infection is shaded yellow. The data show that the concentration of each drug required to inhibit LASV and JUNV pseudovirus infection shifts to the left (i.e., decreases) from a single drug to the two-drug and three-drug combinations. Summary fractional inhibitory concentration (FIC) scores for the drugs against LASV (C) and JUNV (D) are shown. Z-factors for each assay were 0.76 and 0.56, respectively, and the signal-to-noise ratios were 22,546 and 34,059 for LASV and JUNV, respectively. For LASV, the 1× concentrations of Arb, Amo, and Ari were 11, 8, and 12 μM, respectively. For JUNV, the 1× concentrations of Arb, Amo, and Ari were 8, 12, and 5 μM, respectively. Obs, observed; Exp, expected; n/a, not applicable.

**FIG 4:**
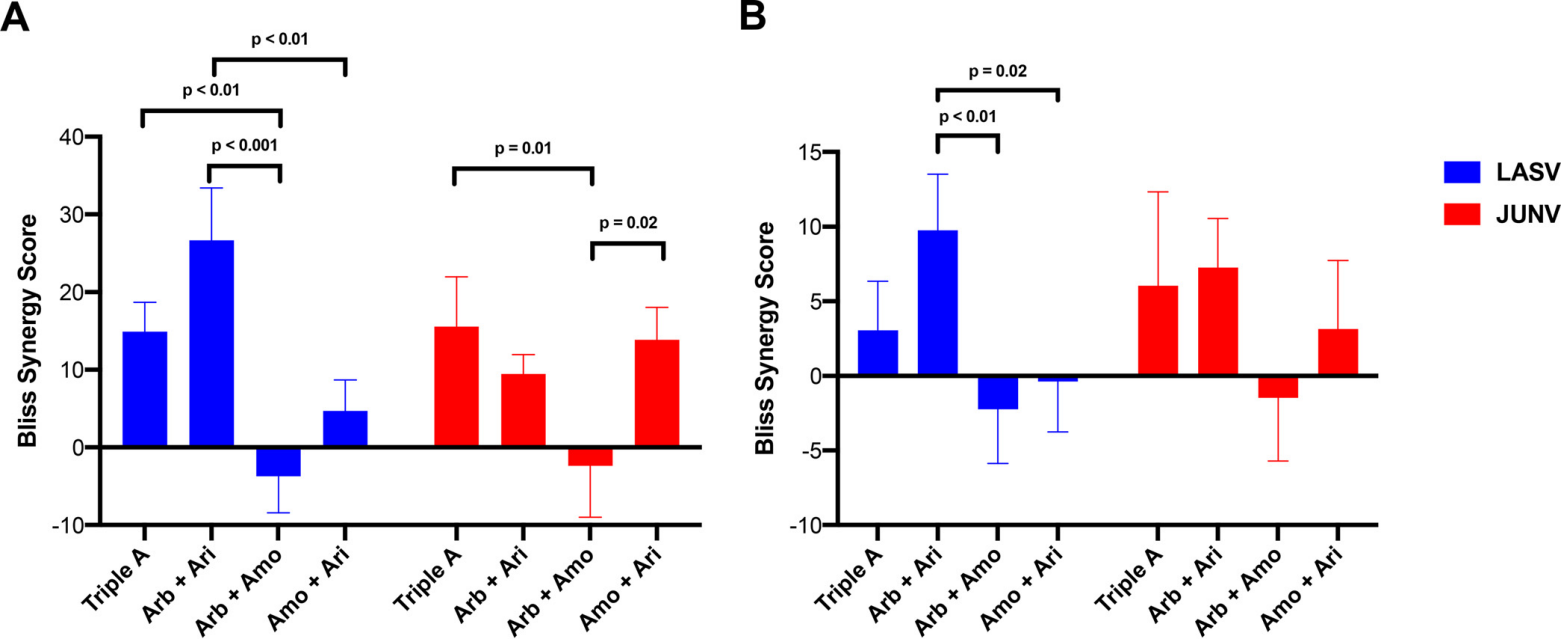
Arbidol synergizes with aripiprazole to suppress arenaviruses. All data were analyzed by SynergyFinder2. Error bars represent the averages of the standard deviations of the overall Bliss synergy scores. (A) Representative pseudovirus results. For LASV, data are derived from a single experiment with a Z score of 0.74 and a signal-to-noise ratio of 33,139. The 1× concentrations of Arb, Amo, and Ari were 11, 8, and 12 μM, respectively. For JUNV, data are derived from a single experiment with a Z score of 0.29 and a signal-to-noise ratio of 34,193. The 1× concentrations of Arb, Amo, and Ari were 8, 12, and 5 μM, respectively. (B) Data reflecting the composite from 8 and 6 separate experiments performed using drug combination assay 1 for LASV and JUNV, respectively. The indicated *P* values are derived from one-way ANOVA using Tukey’s multiple-comparison test in GraphPad Prism. *P* values for all other comparisons were >0.05. Data represent averages and standard deviations from triplicate conditions for each drug combination (A), while the triplicate data in each experiment were averaged across the eight LASV and six JUNV experiments (B).

SynergyFinder2 (51, 52) was then used to further analyze the results from these LASV and JUNV experiments. In individual experiments, the triple-A and arbidol plus aripiprazole combinations were among the most synergistic combinations for both LASV and JUNV, as evidenced by overall Bliss synergy scores of >10 (Fig. 4A). When averaged across all eight experiments, arbidol plus aripiprazole synergistically inhibited LASV, as evidenced by a Bliss synergy score that was significantly higher than those of the other two two-drug combinations (Fig. 4B). For JUNV, the triple-A combination and the combination of arbidol plus aripiprazole showed a trend toward additive to synergistic suppression of pseudovirus infection across all six experiments (Fig. 4B). The combinations of arbidol plus amodiaquine and amodiaquine plus aripiprazole did not confer significant synergy, consistent with the higher FICs produced by this drug combination (Fig. 3). These data suggest that for the three drugs tested, the two-drug combination of arbidol plus aripiprazole caused synergistic suppression of arenaviruses. The addition of amodiaquine to arbidol plus aripiprazole to create the triple-A combination did not lead to significant enhancement of antiviral activity.

**FIG 5:**
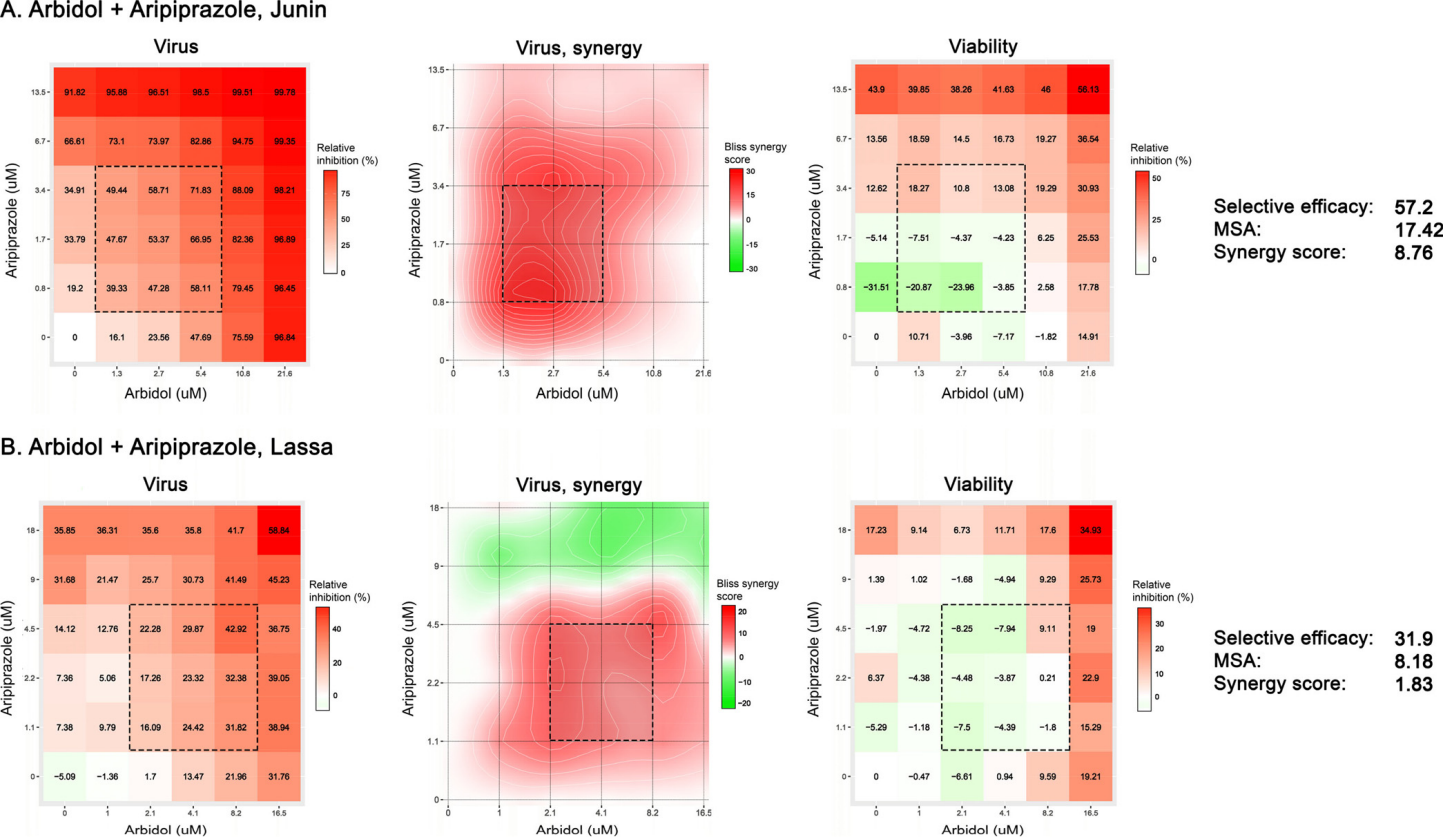
Combining arbidol with aripiprazole causes synergistic suppression of JUNV and LASV pseudoviruses. Vero cells were treated with the indicated concentrations of arbidol and aripiprazole before mock infection or infection with MLV pseudoviruses expressing JUNV (A) or LASV (B) GP; virus infection and cell viability were quantified as described in Materials and Methods. Concentration-response matrices for virus-infected cells were analyzed in SynergyFinder2, which reports the average Bliss synergy score calculated over the full matrix (synergy score) and the maximum Bliss synergy over the maximum synergistic area (MSA) (dotted-line squares). Selective efficacy quantifies the difference between inhibition of virus-infected (virus) and mock-infected (viability) cells. See the text and Materials and Methods for details of these measurements.

Since the combination of aripiprazole plus arbidol appeared synergistic by drug combination assay 1, drug combination assay 2 (i.e., checkerboard assay) was performed in additional experiments with these two drugs, and the results were analyzed with SynergyFinder2. Several parameters were reported from SynergyFinder2, including the average Bliss synergy score of the entire dose-response matrix and the maximum synergistic area (MSA), which corresponds to the maximum Bliss score calculated over an area of 9 doses of the two compounds in a checkerboard experiment (i.e., 3-by-3 dose-response matrix, highlighted by the dotted-line squares in Fig. 5). The selective efficacy quantifies the difference between inhibition of virus-infected (virus) and mock-infected (viability) cells. A selective efficacy of 100 means that the drug combination inhibits 100% of virus-infected cells and does not affect mock-infected, drug-treated cells, while a selective efficacy of 0 means that the drug combination kills 100% of both virus- and mock-infected cells. While there are no established guidelines on what constitutes actual synergy, recent studies suggest that synergy scores of >10 are biologically meaningful (51, 53, 54). Moreover, an analysis of 448,555 anticancer drug combination screens (measured across 124 human cancer cell lines) from the DrugCombDB database (55) reveals that among a full spectrum of drug combination effects, the top 5% of the most synergistic drug combinations exhibit synergy scores of >12 (Fig. S6). Thus, our suggested threshold for synergy (i.e., synergy scores of >10) aligns with the available drug combination data. Figure 5 shows that the combination of aripiprazole plus arbidol conferred synergistic suppression of JUNV and LASV pseudovirus infection, consistent with the FICs and overall Bliss synergy scores from drug combination assay 1. The MSA scores were 17.42 and 8.18 for JUNV and LASV, respectively, indicating that there are specific concentration windows that led to synergistic antiviral effects. Moreover, the MSAs for JUNV and LASV fall within the top 3% and 11% of Bliss synergy scores of large-scale screens (Fig. S6). The selective efficacies of 57.2 and 31.9 for JUNV and LASV pseudoviruses, respectively, indicate strong selective suppression of virus infection but not cell viability.

**FIG 6:**
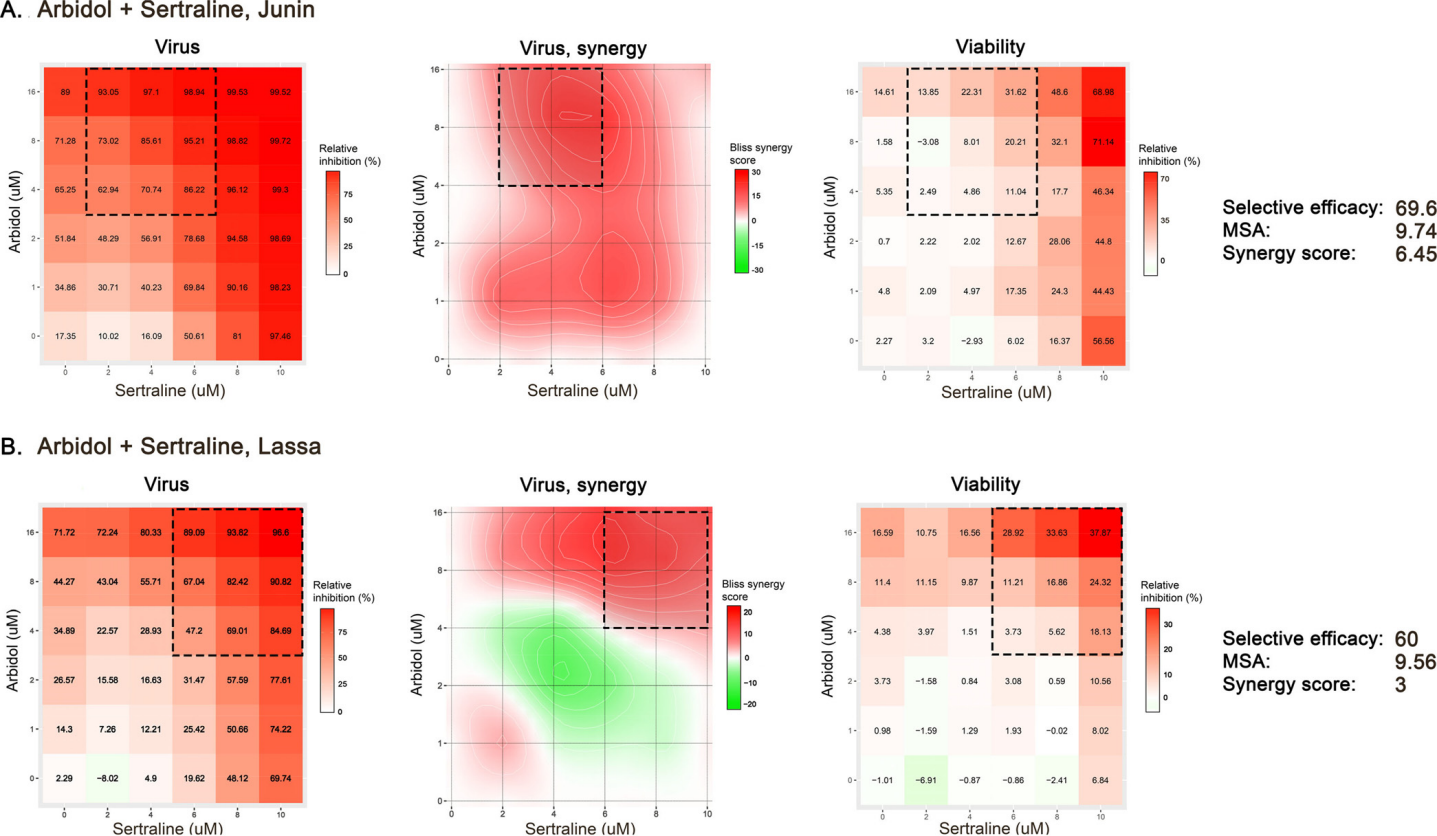
Combining arbidol with sertraline causes synergistic suppression of JUNV and LASV pseudoviruses. Vero cells were treated with the indicated concentrations of arbidol and sertraline before mock infection or infection with MLV pseudoviruses expressing JUNV (A) or LASV (B) GP; virus infection and cell viability were quantified as described in Materials and Methods. Concentration-response matrices for virus-infected cells were analyzed in SynergyFinder2, which reports the average Bliss synergy score calculated over the full matrix (synergy score) and the maximum Bliss synergy over the maximum synergistic area (MSA) (dotted-line squares). Selective efficacy quantifies the difference between inhibition of virus-infected (virus) and mock-infected (viability) cells.

We next evaluated whether arbidol might show synergy when combined with a different approved drug that also acts as a fusion inhibitor. We chose sertraline because it (i) has previously been shown to be a fusion inhibitor for both EBOV and LASV (28), (ii) synergizes with other fusion inhibitors (e.g., toremifene) to suppress EBOV (23, 26, 28, 41, 56, 57), and (iii) inhibits infectious PICV and LASV (Fig. 2; Table S1). SynergyFinder2 analyses of checkerboard assays against JUNV and LASV pseudoviruses showed that arbidol plus sertraline caused modest synergistic suppression of infection, with overall Bliss synergy scores of 6.45 and 3, MSAs of 9.76 and 9.56, and selective efficacies of 69.6 and 60, respectively (Fig. 6). The MSAs for JUNV and LASV fall within the top 8% and 9% of the Bliss synergy scores of large-scale screens (Fig. S6).

The synergistic antiviral effects of sertraline plus arbidol prompted us to evaluate these two drugs with a third drug, aripiprazole, because arbidol plus aripiprazole appeared to be the most synergistic two-drug combination in our three-drug screen (Table 1 and Fig. 3 and 4). All two-drug combinations produced FIC scores of <1 when tested against LASV and JUNV pseudoviruses (Table 2). The synergistic effects occurred at lower concentrations of each drug in the mixture that were not toxic to cells. For LASV, all two-drug combinations appeared to confer similar degrees of synergistic inhibition, while for JUNV, the combination of arbidol plus sertraline seemed to be the most potent two-drug combination. Although the triple-drug combination of arbidol plus sertraline plus aripiprazole conferred the lowest FIC, the Bliss synergy score calculated by SynergyFinder2 for the triple-drug combination was similar to those for the two-drug combinations (data not shown).

**TABLE 2:**
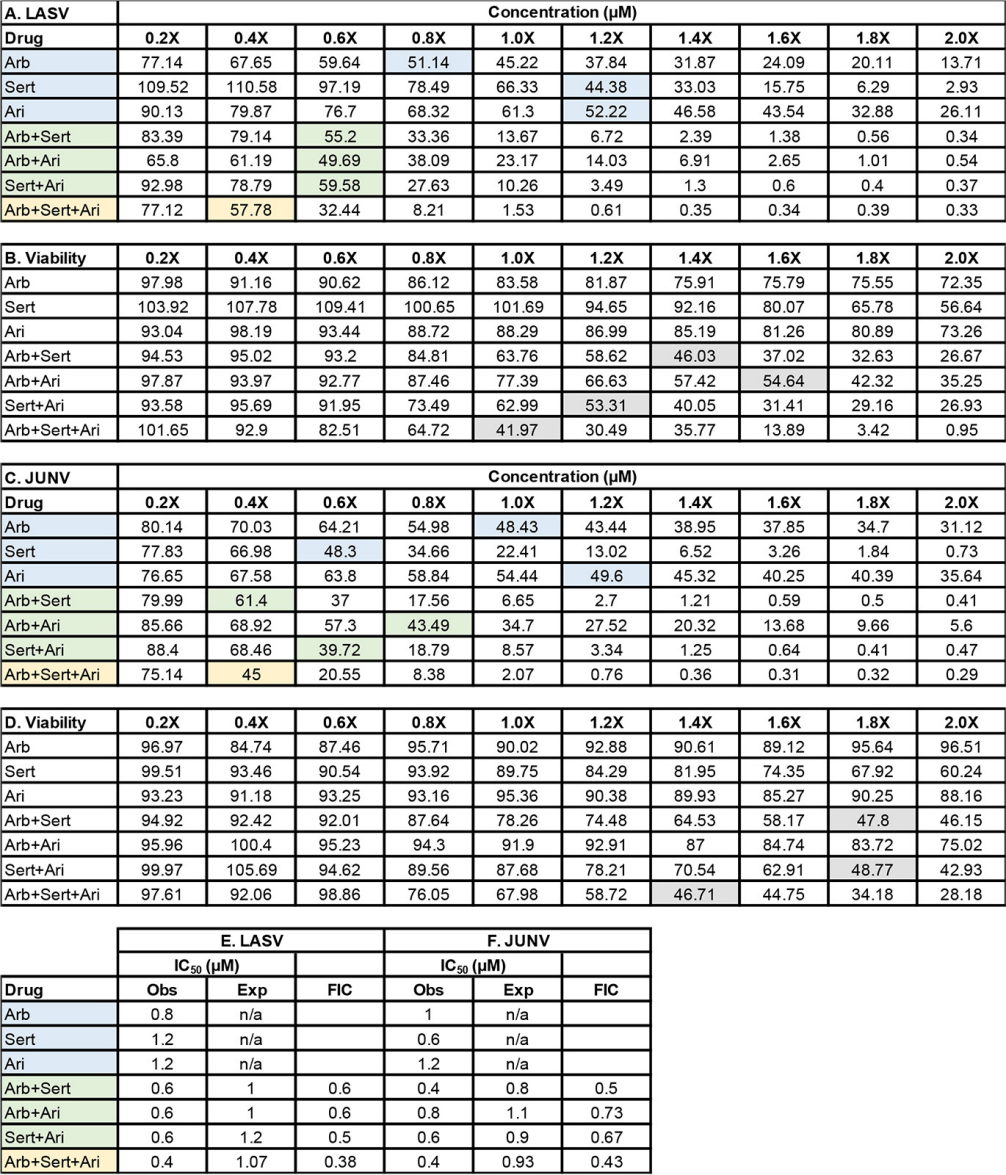
Arbidol combined with the approved drugs aripiprazole and sertraline causes synergistic suppression of LASV and JUNV GP-bearing pseudoviruses^*a*^

a Vero cells were treated with various concentrations of single drugs or a two- or three-drug combination of arbidol (Arb), sertraline (Sert), and aripiprazole (Ari) before infection with LASV or JUNV pseudovirus. Data are expressed as percentages of infected cells treated with the solvent control. At 24 h postinfection, virus infection (A and C) and viability (B and D) were measured. In panels A and C, the concentration of each drug alone providing ∼50% inhibition of infection is shaded blue. The concentration of each drug needed in the pairwise combinations to produce ∼50% inhibition of infection is shaded green. The concentration of each drug needed in the three-drug cocktail to yield ∼50% inhibition of infection is shaded yellow. The gray highlights in panels B and D represent the drug concentrations that inhibit cell viability by approximately 50%. Summary fractional inhibitory concentration (FIC) scores for the drugs against LASV (E) and JUNV (F) are shown. The data are from a single experiment where each condition was conducted in triplicate. For both viruses, the 1× concentrations of Arb, Sert, and Ari were 9 μM. For LASV and JUNV, Z-factors for each assay were 0.71 and 0.52, respectively, while the signal-to-noise values were 27,385 and 335 for LASV and JUNV, respectively. Obs, observed; Exp, expected; n/a, not applicable.

## DISCUSSION

In the current report, the *in vitro* antiviral action of the clinically used anti-influenza virus drug arbidol is confirmed and expanded against filoviruses, including EBOV (23, 32) and MARV, and arenaviruses, including LASV (28), JUNV (28), TACV (32), LCMV, and PICV. We show for the first time that arbidol (a fusion inhibitor), when combined with the approved drug aripiprazole (a macropinocytosis inhibitor) or sertraline (a fusion inhibitor), synergistically inhibits LASV and JUNV pseudovirus infection. Moreover, arbidol, amodiaquine, aripiprazole, sertraline, and niclosamide suppress infectious PICV, while arbidol, sertraline, and niclosamide suppress infectious LASV. This report provides further proof of concept for the potential of repurposing combinations of approved, orally available drugs for viral outbreak preparedness and control (26, 27). Further study is required to identify additional approved drugs that yield more potent two- or three-drug combinations that further enhance antiviral synergy compared to two-drug combinations. Indeed, a recent study described several compounds with potent broad-spectrum antiviral activity (58), which may enhance antiviral synergy against arenaviruses (59).

How do the *in vitro* IC_50_ values relate to concentrations achieved in human plasma and tissues following oral administration of these drugs? The recommended dosage of arbidol for the treatment of influenza is 200 mg orally three to four times daily for 5 days, and longer durations of treatment of up to 20 days have been reported (34). The same frequency and varying durations are currently in use for coronavirus disease 2019 (COVID-19) (38, 60, 61), and single doses of up to 800 mg have been administered without adverse effects (62). The maximum plasma concentration (*C*_max_) after a single oral 200-mg dose in humans ranges from 0.9 to 1.5 μM (62–64), which is lower than the *in vitro* IC_50_ values reported in this work when arbidol is given as a monotherapy against arenaviruses (6 to 10 μM) (Fig. 1 and 2; see also Table S1 in the supplemental material). For filoviruses, arbidol monotherapy suppresses EBOV and MARV with *in vitro* IC_50_s of 2.7 to 3.9 μM (Fig. S3) (28, 32). However, a single 800-mg dose elicits a *C*_max_ of 4 μM (62), and with repeated daily dosing over several days, drug accumulation in plasma and tissues infected by viruses may occur. In this report, combining arbidol with aripiprazole or sertraline reduced the IC_50_ of arbidol ∼2-fold to 3 to 4 μM (Tables 1 and 2), a concentration achievable in plasma following oral administration of arbidol. For aripiprazole, the *C*_max_ in humans ranges from 0.17 to 1.0 μM upon the administration of 5 to 30 mg daily for 14 days (65). However, *C*_max_ values of 2 to 5 μM after 14 days have been reported (66), which are near the *in vitro* IC_50_ values for aripiprazole (4.5 to 6.0 μM) that synergized with arbidol or sertraline to suppress LASV and JUNV infection. For sertraline, the *C*_max_ ranges from 0.07 to 0.18 μM after a single 25- to 100-mg dose (67), while after 21 days of a 200-mg daily dose, the *C*_max_ ranges from 0.39 to 0.54 μM (68). Thus, the *in vitro* IC_50_ of sertraline (3.6 to 5.4 μM) that synergized with arbidol or aripiprazole to suppress LASV and JUNV infection is currently higher than what can be achieved *in vivo*. Future studies should identify additional approved drugs that synergize with arbidol plus aripiprazole and arbidol plus sertraline to further reduce the *in vitro* IC_50_s of all drugs to concentrations at or below the *C*_max_. This therapeutic strategy could minimize unwanted pharmacokinetic and/or pharmacodynamic drug-drug interactions. For more robust *in vitro-in vivo* comparisons, additional pharmacokinetic factors must be considered, including blood-to-tissue partitioning, plasma protein and tissue binding, the accumulation ratio upon multiple dosing, and the effects of infection. Future *in vitro* screening of drug combinations, coupled with clinical pharmacology- and computation-informed selection and triage criteria, may provide lead combinations for rapid advancement to *in vivo* animal and possibly clinical testing. In this regard, compared to BSL3 or BSL4 live-virus work, BSL2-compatible pseudovirus systems confer easy and affordable approaches for higher-throughput screening and testing of virus entry inhibitors. Upon the identification of synergistic approved drug combinations with pseudoviruses, combination studies with postentry inhibitors can be performed against live-virus infections.

Most of the current drug countermeasures for emerging and reemerging acute viral infections are single agents, yet all successful antiviral therapies for chronic viral infections are based on combinations of drugs (69, 70). A stockpile of orally available, room-temperature-stable combinations of approved drugs that synergistically inhibit infections by arenaviruses, filoviruses, and possibly members of other virus families at multiple stages of infection could reduce viral loads, virus-induced inflammation (e.g., cytokine storms), pathogenesis, and case fatality rates. Indeed, a recent outbreak of the arenavirus Chapare virus (CHAPV) in Bolivia (71), which affected health care workers, may benefit from such an approach. Such drug combinations could provide coverage when new strains arise that are not covered by available vaccines, therapeutic antibodies, and/or drugs directed against specific viral proteins. Moreover, targeting different stages of infection would reduce the likelihood of the emergence of drug-resistant strains (69, 70). Stochastic models have shown that aggressive deployment of antiviral medications can curtail an outbreak (72), and World Health Organization guidelines state that “unless a country has a stockpile, it will not have antivirals available to use in a pandemic,” the situation that we are currently facing with the SARS-CoV-2 pandemic. Because the development, safety and efficacy testing, scale-up, and deployment of vaccines, therapeutic antibodies, and designer antiviral drugs are cost- and time-intensive, a stockpile of approved oral drugs/drug combinations with activity against related virus family members could be invaluable as a first line of defense to reduce virus transmission and related morbidities and mortalities during the initial waves of pandemic infections.

In summary, arbidol and several other approved drugs inhibit multiple arenaviruses, and when arbidol is combined with other approved drugs, the drug combinations exert synergistic suppression of arenaviruses. Our findings provide further proof of concept that repurposing combinations of approved oral drugs is a proactive way forward for global preparation as a rapidly deployable, first line of defense for future virus outbreaks and perhaps even for the current SARS-CoV-2 pandemic. In this regard, approved-drug screens have shown that the same drugs inhibit EBOV (23, 32), LASV (28), SARS and Middle East respiratory syndrome (MERS) coronaviruses (73), SARS-CoV-2 (38, 43, 74, 75), and many other viruses (58). Repurposing of approved drugs in carefully tested and validated combinations may offer a proactive new strategy for controlling known and new viral outbreaks in the future through (i) cost reductions in antiviral drug development, (ii) application to other medically significant viruses that share similar routes of entry into cells, (iii) enhanced outbreak readiness through stockpiling without a need for cold-chain storage, and (iv) affordability for global deployment.

## MATERIALS AND METHODS

### Chemicals, cell culture, and live virus.

Vero, Vero E6, and 293T cells were maintained in standard medium (Dulbecco’s modified Eagle medium [DMEM] [catalog number 11995-065; Gibco] supplemented with 9% fetal bovine serum [FBS] [catalog number 16000-044; Gibco] and 1% penicillin-streptomycin [catalog number 15140-122; Gibco]). Arbidol (Arb) was synthesized commercially, and the purity and structure of the product were confirmed as described previously (32). Amodiaquine (Amo) was purchased from Sigma-Aldrich (catalog number A2799-5G), and aripiprazole (Ari) and sertraline were purchased from Selleckchem (catalog numbers S1975 and S4053, respectively). Infectious Pichinde virus (PICV-GFP) was a recombinant virus, rP18tri-GFP, that expresses GFP as a reporter (48). The parental virus was generated from passage 18 of PICV that produces Lassa-like disease in guinea pigs (76). The Lassa virus (Josiah) strain was propagated as described previously (77).

### Production of pseudovirus.

Murine leukemia reporter viruses pseudotyped with glycoproteins (GPs) from filoviruses (EBOV [Zaire Mayinga isolate] [GP plasmid provided by Erica Sapphire] and MARV [Angola and Musoke isolates] [GP plasmids provided by Chris Broder]) and arenaviruses (LASV [Josiah isolate] [GP plasmid provided by Gregory Melykian], JUNV, LCMV [GP plasmid provided by Jack Nunberg], and PICV [wild type {WT} and R55A mutant {47}]) were generated as follows. 293T cells were seeded in a T175 flask (catalog number 431080; Corning) in transfection medium (DMEM without phenol red [catalog number 31053036; Gibco], 9% FBS, 1% l-glutamine [catalog number 25030-081; Gibco], 1% sodium pyruvate [catalog number 11360-070; Gibco]) and incubated at 37°C with 5% CO_2_ overnight. The next day, 1,000 μl of Opti-MEM (catalog number 11058-021; Gibco) was added to a 1.5-ml tube, followed by 55.2 μl of X-tremeGENE 9 DNA transfection reagent (catalog number 06 365 787 001; Roche Applied Science). Plasmids pTG-Luc (7.4 μg) (provided by Jean Dubuisson), pCMV-MLV (gag-pol) (3.7 μg) (provided by Jean Dubuisson), gag-BlaM (3.7 μg), and the respective viral fusion GP (3.7 μg) were then added to the tube. After a quick vortex and a brief spin, transfection mixtures were incubated for 15 min at room temperature, followed by another quick vortex and spin. Complexes were then added to the 293T cells previously seeded into T175 flasks and incubated at 37°C with 5% CO_2_ for 48 h. Following incubation, each pseudovirus (PV) preparation was harvested by adding medium from the T175 flask to two 15-ml Falcon tubes (catalog number 352196; Corning), followed by centrifugation at 800 × *g* at 4°C for 7 min to pellet cell debris. Aliquots (1 ml) of each PV stock were stored at −80°C, and freeze-thaws were avoided.

### Detection of viral glycoproteins.

Viral GPs were detected by Western blotting using an arenavirus-specific monoclonal antibody (22.5D; Zalgen Labs) (provided by Luis Branco and Robert Garry), an Ebola virus-specific monoclonal antibody (H3C8) (hybridomas kindly provided by Caroline Wilson), and a rabbit anti-MARV GP polyclonal antibody (catalog number 0303-007; IBT Bioservices). MLV p30 gag protein was detected with a mouse monoclonal anti-MLV p30 antibody (4B2) (catalog number ab130757; Abcam). The GPs were detected in nonconcentrated pseudovirus stocks. Western blot analyses were performed as described previously (78).

### Infection of cells with pseudovirus or live virus.

Pseudovirus stocks were thawed and allowed to come to room temperature. Using a multichannel pipette, 100 μl of the virus stock was added to each well of columns 2 to 12 on three 96-well plates. Column 1 on each plate served as the mock-infected control: 100 μl of standard medium was added to each well in column 1. Plates were then spun at 300 × *g* for 1 h at 4°C, after which the plate was taken to a biosafety cabinet and the lid was removed for 10 to 15 s to evaporate the condensate on the lid. The plate was then incubated at 37°C with 5% CO_2_ for 24 h. For experiments with arbidol, Vero cells were pretreated with the six to eight different drug concentrations prior to infection of cells with pseudovirus as described previously (28). For testing of arbidol against PICV-GFP, Vero E6 cells were pretreated with various drug concentrations for 1 h prior to infection with PICV-GFP at a multiplicity of infection (MOI) of 0.1. For LASV Josiah infections, Vero E6 cells were treated with various concentrations of drugs for 1 h prior to infection with LASV Josiah at an MOI of 0.2, and a cell-based enzyme-linked immunosorbent assay (ELISA) was used to quantify viral infectivity (77; L. DeWald, E. M. Morazzani, L. M. Johansen, L. T. Pierce, J. M. Grenier, B. M. Friedrich, C. M. Lear-Rooney, A. E. Piper, A. R. Stossel, C. J. Fitzpatrick, S. M. Stronsky, G. G. Olinger, and P. J. Glass, unpublished data). For both viruses, all conditions were conducted in triplicate.

### Fluorescence microscopy and quantitation for PICV-GFP.

At 48 h postinfection, medium was removed from PICV-GFP-infected Vero E6 cultures, and cells were washed in phosphate-buffered saline (PBS) and fixed in 4% paraformaldehyde for 20 min at room temperature. Cells were then washed twice in PBS and permeabilized in 0.3% Triton X-100 in PBS for 15 min. Fixed cells were then stained with 4′,6-diamidino-2-phenylindole (DAPI) and imaged using a Cytation 1 cell imaging system (BioTek, Winooski, VT), using a 10× objective and light cubes for DAPI (nuclear stain) and GFP (infection reporter). Gen5 software (BioTek) was used for image acquisition, processing, and subsequent analysis. The 10× objective was used to take 64 individual images using an 8-by-8 matrix, which were stitched together utilizing Gen5’s montage feature. The DAPI threshold was set at 4,000 relative fluorescence units and the GFP threshold was set at 900 relative fluorescence units to identify nuclei and infected cells, respectively. Analysis identified nuclei in the DAPI channel utilizing minimum and maximum size selections of 8 μm and 35 μm, respectively, to capture only stained nuclei. GFP-positive cells were identified utilizing minimum and maximum size selections of 15 μm and 58 μm, respectively, to capture entire GFP-positive cells.

### Cell viability assays.

For approved-drug dose-response studies, we measured cell viability in parallel plates using the ATPlite kit (catalog number 6016943; PerkinElmer). For most drug combination assays, we used PrestoBlue (PB) HS cell viability reagent (catalog number P50201; Invitrogen) to measure cell viability in the same wells as for virus-produced luciferase measurements. The assay detects the reduction of resazurin to a red fluorescent dye within the reducing environment of viable cells. This change can be measured by the absorbance using 600-nm and 570-nm wavelengths. Briefly, PB was added to wells to 11.1% of the well volume and incubated at 37°C with 5% CO_2_ for 2 h. The plate was then read in a Molecular Devices Spectra Max Plus 384 plate reader at 570 nm and 600 nm. The 600-nm readings were subtracted from the 570-nm readings, followed by subtraction of the average of the medium-only background wells. Replicates were averaged across plates and divided by the average of the solvent plus PV control wells to obtain the fraction of the control. Results were multiplied by 100 to obtain the percentage of the control. Next, the contents of each well were aspirated, and 50 μl of PBS (catalog number 10010031; Gibco) was added to each well. Fifty microliters of Britelite reagent (catalog number 6066761; PerkinElmer) was added to each well. The plate was then placed for approximately 10 min on low on a plate shaker and then read for luminescence on a Victor plate reader (PerkinElmer).

### Drug combination assay 1.

For drug combination assay 1, we adapted the protocol of Cokol-Cakmak et al. (50). This method allows three different drugs to be tested individually, in three two-drug combinations, and as a triple combination on a single 96-well plate over a range of 10 uniformly divided concentrations. For each set of three drugs, three 96-well plates were used to provide triplicate conditions. Each row of a 96-well plate contained the three single drugs (rows A, B, and C), the three two-drug combinations (rows D, E, and F), and the triple-drug combination (row G). The eighth and final row, row H, contained the solvent control, a 1:1 mixture of dimethyl sulfoxide (DMSO)-ethanol, at a concentration of 1% in all wells. The final concentration of DMSO and ethanol was 0.5%.

The experimental setup involves 4 steps: (i) defining the stock concentration of each drug, (ii) preparing the drug master mix plate for the seven drug combinations (three single drugs, three two-drug combinations, and one three-drug combination) plus one solvent control, (iii) preparing the drug dilution plate, and (iv) transferring 2 μl of drugs from the drug dilution plate to the three plates of cells. These steps are summarized below and schematized in Fig. S4 in the supplemental material. The concentrations of the drugs tested decreased equally by 10% across the dilution series (Fig. S4D). Thus, single drugs and drug combinations were tested over a concentration range of 0.125 to 1.25×, with × representing the IC_50_ of the drug(s) in question. The concentration range was divided into equal intervals of 10% and plated in columns 2 through 11 of the 96-well plate. Specifically, concentrations of 0.125, 0.25, 0.375, 0.5, 0.625, 0.75, 0.875, 1.0, 1.125, and 1.25× the IC_50_ were tested for the single drugs and drug combinations. For drug combinations, each drug was tested at its separately determined IC_50_. This method is adaptable in terms of (i) the concentration range tested and (ii) how the concentration range is partitioned into 10 equal intervals.

Five thousand Vero cells were seeded into each well of three 96-well, sterile, tissue-culture-treated, black, clear-bottom plates (catalog number 6005182; PerkinElmer) in a final volume of 98 μl of standard medium per well. The three plates were incubated at 37°C with 5% CO_2_ overnight. Stock solutions of each drug (A, B, and C) were made at 437.5× their previously determined IC_50_ in a 1:1 mixture of ethanol-DMSO solvent (200-proof ethanol from Decon Labs [CAS number 64-17-5] and DMSO from Mediatech [catalog number 25-950-CQC]), and drug stocks were vortexed thoroughly. Two hundred microliters of each drug stock (A, B, and C) was added to 500 μl of solvent in separate sterile 1.5-ml Eppendorf tubes to generate 125× stocks of each single drug (Fig. S4A). To generate stocks of two-drug mixtures, 200 μl of drugs A and B (A+B) (each at 437.5×) was added to 300 μl of the solvent in one 1.5-ml tube, yielding a 125× stock of drugs A+B. Two-drug mixtures of drugs A+C and drugs B+C were similarly generated (Fig. S4A). To generate the three-drug mixture, 200 μl of drugs A, B, and C (each at 437.5×) was added to 100 μl of the solvent in one 1.5-ml tube, yielding a 125× stock of drugs A+B+C (Fig. S4A). The solvent control tube was generated by transferring 700 μl of the solvent to a separate 1.5-ml tube (Fig. S4A). These eight tubes were vortexed, followed by a quick spin. Next, the contents of these eight tubes were separately pipetted into their own well in column 1 in a deep-well, sterile, non-tissue-culture-treated, clear 96-well plate to generate the drug master mix plate (Fig. S4A). From this plate, for each single-, double-, or triple-drug mixture, 100, 90, 80, 70, 60, 50, 40, 30, 20, 10, and 0 μl were pipetted horizontally (i.e., across the respective rows) into each well of columns 2 through 11 of a separate sterile, clear 96-well plate using a multichannel pipette to yield the drug dilution plate (Fig. S4B). Next, the solvent was pipetted across columns 2 through 11 at volumes of 0, 10, 20, 30, 40, 50, 60, 70, 80, 90, and 100 μl. The drug dilution plate therefore represents the concentration range divided into equal intervals as described above, with the final concentrations of the single-, double-, and triple-drug mixtures being 12.5, 25, 37.5, 50, 62.5, 75, 87.5, 100, 112.5, and 125×. The lid was placed on the plate, which was placed on a plate shaker on the low setting for 10 min at room temperature. Using a multichannel pipette, 2 μl from each well on the drug dilution plate was transferred to its corresponding well on one of the three plates of Vero cells plated the day before (Fig. S4C). This final 1:100 dilution yielded the desired concentration range of 0.125 to 1.25× divided into 10 equal intervals (Fig. S4D).

### Drug combination assay 2.

For drug combination assay 2, 6-by-6 checkerboard experiments were performed (i.e., two drugs were tested at all possible combinations for 36 combinations of two drugs). Five thousand Vero cells were seeded into each well of two 96-well, black, clear-bottom plates in a final volume of 98 μl of standard medium per well. The two plates were incubated at 37°C with 5% CO_2_ overnight. Stock solutions of each drug (A and B) were made at 400× their previously determined IC_50_s in 1,000 μl of a 1:1 mixture of ethanol-DMSO solvent, in separate sterile 1.6-ml Eppendorf tubes, and drug stocks were vortexed thoroughly. Five hundred microliters of each drug stock (A and B) was added to 500 μl of the solvent in separate sterile 1.6-ml Eppendorf tubes. This step was serially repeated four times to generate five concentrations of each drug, with 2-fold differences between each concentration. A final 1.6-ml Eppendorf tube was filled with 500 μl of the solvent. The dilution series for each drug and solvent were added to their own row of a deep-well, sterile, non-tissue-culture-treated 96-well plate. In a sterile, non-tissue-culture-treated 96-well drug dilution plate, 100 μl of the solvent was pipetted into the first half of the top row (wells A1 to A6) and the first half of the bottom row (wells H1 to H6) of the plate. Fifty microliters from the row in the deep-well plate containing the serial dilutions of drug A was pipetted into wells B1 to B6 in the drug dilution plate. This procedure was repeated for rows C to G. The drug dilution plate was turned clockwise 90°, and 50 μl from the row in the deep-well plate containing the serial dilutions of drug B was pipetted into columns B1 to G1 in the drug dilution plate. This procedure was repeated for columns 2 to 6, e.g., B2 to G2 and B3 to G3, etc., to complete the 6-by-6 checkerboard. The lid was placed on the plate, which was then placed on a plate shaker on the low setting for 10 min at room temperature. Using a multichannel pipette, 2 μl from each well on the drug dilution plate was transferred to the two cell plates. Each 96-well plate allowed plating of two 6-by-6 checkerboards per plate. Thus, testing of each two-drug concentration in the 6-by-6 checkerboard was performed in quadruplicate.

### Data and drug combination analyses.

For single-drug experiments with arbidol, drug concentrations were log transformed, and the concentration of the drug(s) that inhibited virus by 50% (i.e., IC_50_) and the concentration of the drug(s) that killed 50% of cells (i.e., CC_50_) were determined via nonlinear logistic regressions of log(inhibitor) versus response-variable dose-response functions (four parameters) constrained to the zero-bottom asymptote by statistical analysis using GraphPad Prism 9 (GraphPad Software, Inc.), as described previously (28). The selectivity index was calculated by dividing the CC_50_ by the IC_50_.

For drug combination assay 1, the Z-factor was calculated as follows:

$$Z\prime =1\;-\;\frac{3\left({\hat{\sigma }}_{p}\;+\;{\hat{\sigma }}_{n}\right)}{\left|{\hat{\mu }}_{p}\;-\;{\hat{\mu }}_{n}\right|}$$

where σ^ indicates sample standard deviations, μ^ indicates sample means, subscript *p* indicates the positive control, and subscript *n* indicates the negative control. Data presented here include data from LASV and JUNV pseudovirus infection experiments with average Z-factors of 0.71 ± 0.06 and 0.5 ± 0.11, respectively.

Fractional inhibitory concentration (FIC) scores were calculated by dividing the observed IC_50_ by the expected IC_50_ for each two-drug and three-drug combination. The expected IC_50_ for the two- or three-drug combinations was calculated by the arithmetic mean of the IC_50_ for each drug tested individually. By examining the single-drug dose-response data, the concentration closest to 50% inhibition was designated the observed IC_50_. FICs of 1 suggest additivity, FICs of >1 suggest antagonism, and FICs of <1 suggest synergism (49, 50). Data from multiple experiments were analyzed by one-way analysis of variance (ANOVA) using Tukey’s multiple-comparison test in GraphPad Prism 9.

Data from three-drug combination (drug combination assay 1) and two-drug checkerboard (drug combination assay 2) tests were analyzed in SynergyFinder2, an open-access platform for multidrug combination synergies (51, 52). For the combination synergy model, we used the Bliss independence model, which assumes a stochastic process in which the drugs elicit their effects independently, and the expected combination effect can be calculated based on the probability of independent events (79). Several parameters were reported from SynergyFinder2, including the average Bliss synergy score of the entire dose-response matrix and the maximum synergistic area (MSA), which corresponds to the maximum Bliss score calculated over an area of 9 doses of the two compounds in a checkerboard experiment (i.e., 3-by-3 dose-response matrix). The selective efficacy was calculated as the average percent viability difference between efficacy (viability of virus-infected cells) and toxicity (viability of control cells). Selective efficacy quantifies the difference between inhibition of virus-infected (virus) and mock-infected (viability) cells. A selective efficacy of 100 means that the drug combination inhibits 100% of virus-infected cells and does not affect mock-infected cells, while a selective efficacy of 0 means that the drug combination inhibits 100% of both virus- and mock-infected cells.

## Supplementary Material

Supplemental file 1
