# Supplementary material for: Inhibition of Arenaviruses by Combinations of Orally Available Approved Drugs
Source: Antimicrob Agents Chemother. 2021 Mar 18;65(4):e01146-20. doi: 10.1128/AAC.01146-20 (PMC8097473; doi:10.1128/AAC.01146-20)
Supplement: Supplemental file 1 [file AAC.01146-20-s0001.pdf]

Supplemental Material FOR Publication

Approved Drug	IC_{50} PICV^	IC_{50} LASV
Arbidol	8.6	~10.0#
Amodiaquine	4.5	nt
Aripiprazole	5.4	nt
Sertraline	3.7	7.0*
Niclosamide	<0.2	0.2*

TABLE S1: Inhibition of infectious PICV and LASV by multiple approved drugs. The Table shows IC_{50} values for the indicated drugs tested against PICV-GFP in this study^ and against LASV Josiah as described in (1)* and (2)#. nt = not tested. For PICV-GFP, Vero E6 cells were treated with varying concentrations of drugs for 1 hour prior to infection with PICV-GFP at a MOI of 0.1. Forty-eight hours post-infection, cells were fixed, counterstained with DAPI, and GFP fluorescence measured on a Cytation 1 imaging system. Data represent averages of 4, 1, 2, 3, and 3 experiments for arbidol, amodiaquine, aripiprazole, sertraline, and niclosamide, respectively. For LASV, Vero E6 cells were treated with varying concentrations of drugs for 1 hour prior to infection with LASV Josiah at an MOI of 0.2. For both viruses, all conditions were conducted in triplicate.

SUPPLEMENTAL FIGURE LEGENDS

Fig S1: Approved drugs evaluated for inhibition of arenaviruses. Upon binding to cells, many enveloped viruses internalize and move into the cell (*i.e.*, traffic) to an acidic endosome. To deliver their genomes into cells, enveloped viruses must then fuse their membranes with the membrane of an endosome. As examples, the viral glycoproteins (GPs) of arenaviruses and filoviruses mediate the membrane fusion event (3), which is primed and triggered in the acidic environment of the endosome. The drugs tested in this study (aripiprazole, amodiaquine, niclosamide, arbidol, and sertraline) inhibit virus entry by acting at distinct steps of virus entry.

Fig S2: Expression of arenavirus and filovirus glycoproteins in MLV pseudovirus stocks. Viral GP expression was tested on non-concentrated pseudovirus stocks. Antibodies used are described in the Materials and Methods.

Fig S3: Arbidol inhibits infection of MLV pseudoviruses bearing filovirus glycoproteins from MARV. Vero cells were treated with varying concentrations of arbidol prior to infection with MLV pseudoviruses that enter cells via the glycoproteins of MARV Angola or MARV Musoke. Twenty-four hours later, luciferase activity was measured to quantify virus infection, and ATP levels were measured to quantify cell viability. Error bars represent standard deviations. Each condition was performed in triplicate, and each experiment was performed three times for each MARV pseudovirus.

For each virus, the data depict the averages and standard deviations across all experiments performed for that virus.

Fig S4: Testing Setup for Drug Combination Assay 1. See Materials and Methods for details of the procedure. Mock = mock-infected cells. Sol = solvent control. X = a defined µM concentration of one-, two-, or three-drug combinations.

Fig S5. Synergistic inhibition of MLV pseudoviruses bearing LASV and JUNV glycoproteins. Dose-response curves (average values +/- SD) for the data presented in **Figs 3, 4** and **Table 1**.

Fig S6. Distribution of drug combination Bliss synergy scores. 448,555 drug combinations (measured across 124 human cancer cell lines) were extracted from the DrugCombDB database (4), and Bliss Synergy Scores were calculated for each drug combinations. It was assumed that approximately 5% of all the combination experiments accounted for the most synergistic scores, and 5% confer the most antagonistic scores. The average synergy scores were quantiled (%) as follows: 5% = 12.3; 10% = 8.4; 25% = 3.8; 50% = 0.2; 75% = -3.3; 95% = -16.5.

REFERENCES

1. DeWald L, E.M. Morazzani, L.M. Johansen, L.T. Pierce, J.M. Grenier, B.M. Friedrich, C.M. Lear-Rooney, A.E. Piper, A.R. Stossel, C.J. Fitzpatrick, S.M. Stronsky, G.G. Olinger, and P.J. Glass. 2021. Identification of FDA-approved drugs with broad activity against high-consequence viral pathogens. manuscript in preparation.

2. Hulseberg CE, Feneant L, Szymanska-de Wijs KM, Kessler NP, Nelson EA, Shoemaker CJ, Schmaljohn CS, Polyak SJ, White JM. 2019. Arbidol and Other Low-Molecular-Weight Drugs That Inhibit Lassa and Ebola Viruses. J Virol 93.
3. White JM, Whittaker GR. 2016. Fusion of Enveloped Viruses in Endosomes. Traffic 17:593-614.
4. Liu H, Zhang W, Zou B, Wang J, Deng Y, Deng L. 2020. DrugCombDB: a comprehensive database of drug combinations toward the discovery of combinatorial therapy. Nucleic Acids Res 48:D871-D881.

Fig S1

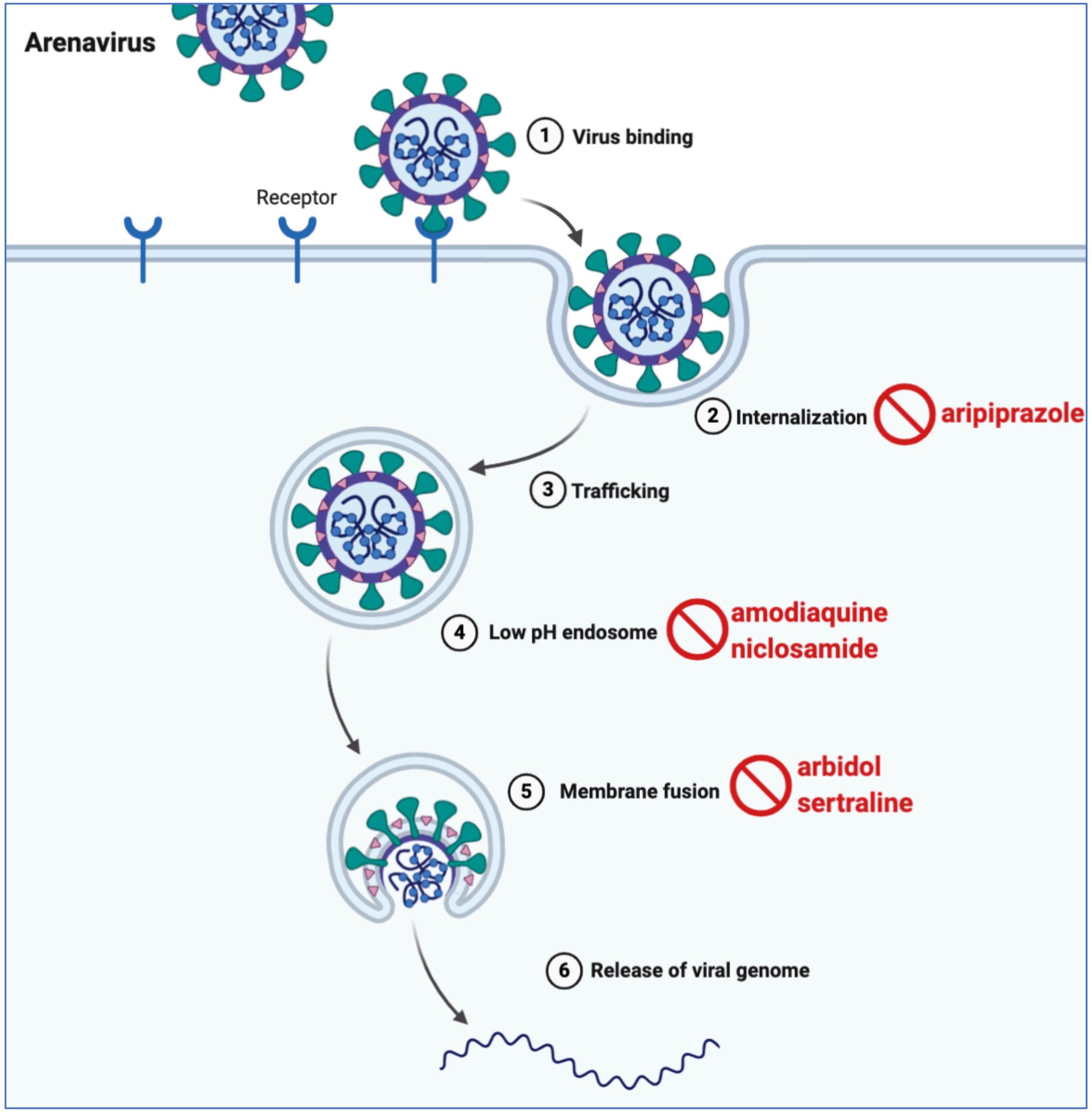
Arenavirus
1 Virus binding
Receptor
2 Internalization
aripiprazole
3 Trafficking
4 Low pH endosome
amodiaquine
niclosamide
5 Membrane fusion
arbidol
sertraline
6 Release of viral genome

Fig S2

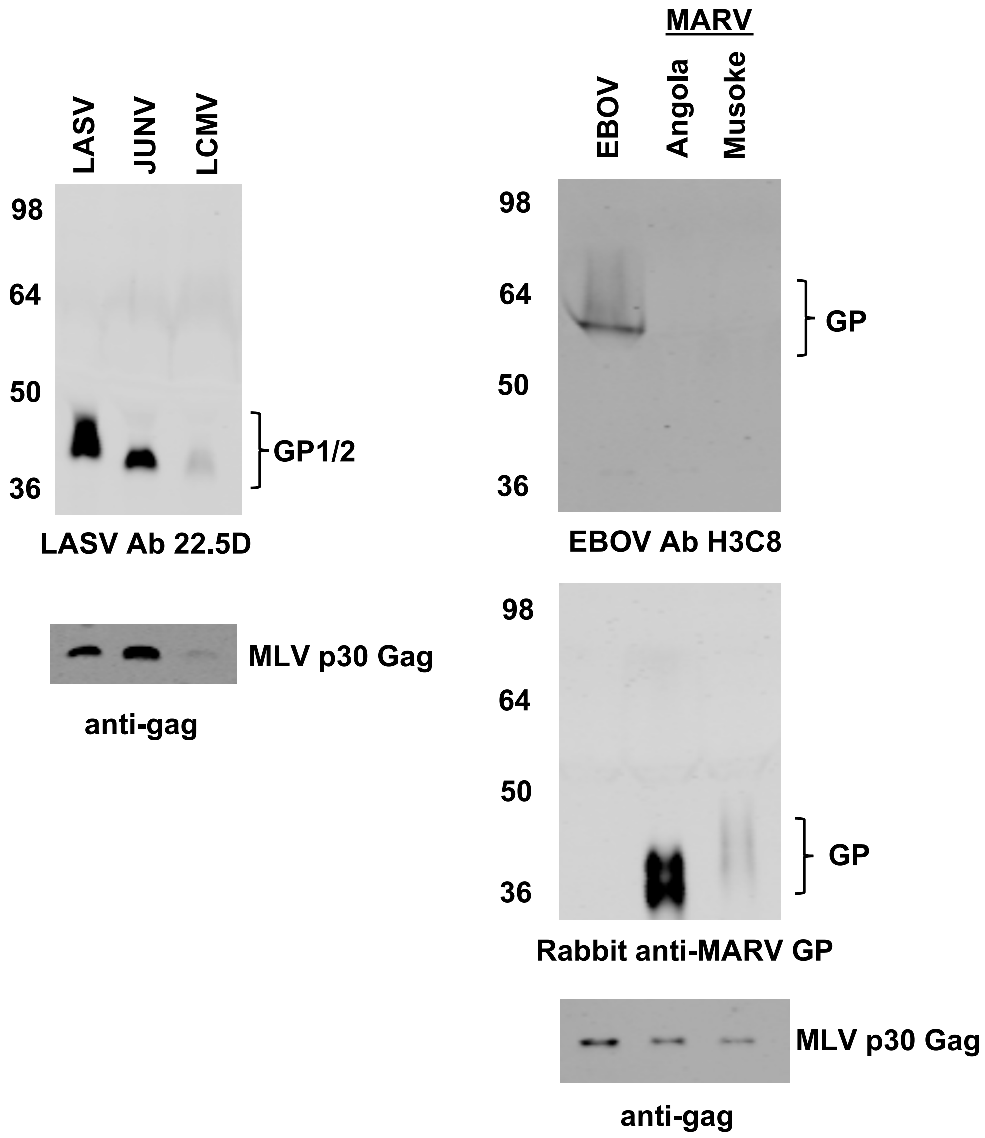
LASV
JUNV
LCMV
98
64
50
36
GP1/2
LASV Ab 22.5D
MLV p30 Gag
anti-gag
MARV
EBOV
Angola
Musoke
98
64
50
36
GP
EBOV Ab H3C8
98
64
50
36
GP
Rabbit anti-MARV GP
MLV p30 Gag
anti-gag

Fig S3

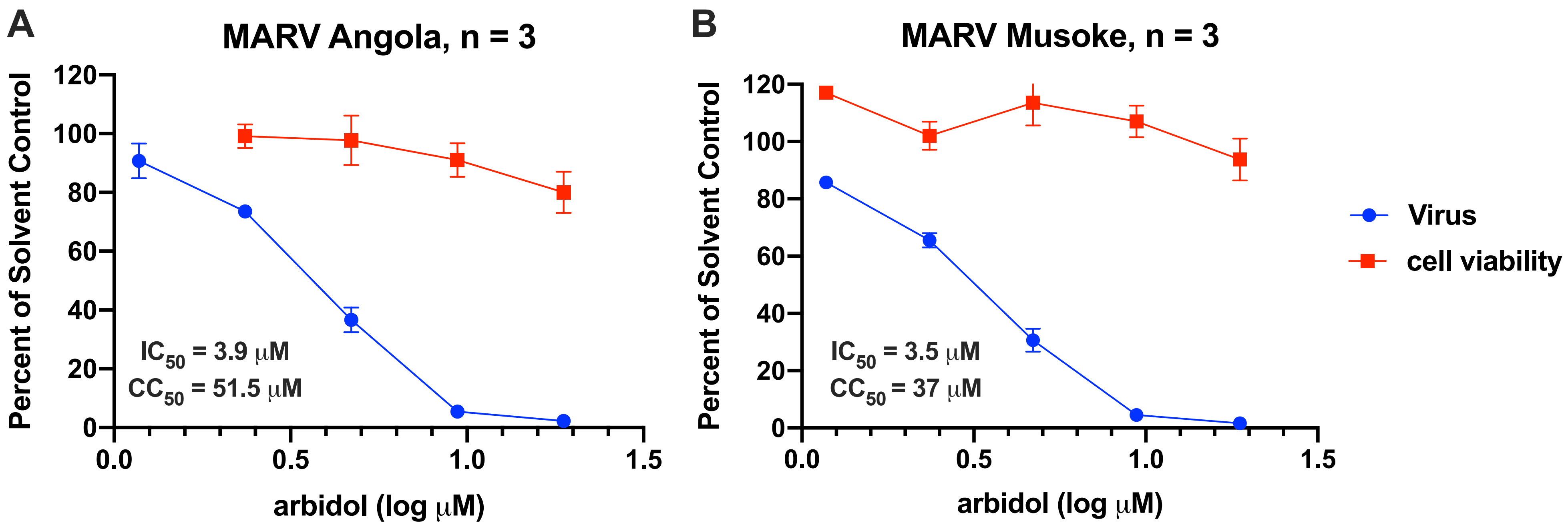
A
MARV Angola, n = 3
Percent of Solvent Control
120
100
80
60
40
20
0
0.0
0.5
1.0
1.5
arbidol (log μM)
IC50 = 3.9 μM
CC50 = 51.5 μM
B
MARV Musoke, n = 3
Percent of Solvent Control
120
100
80
60
40
20
0
0.0
0.5
1.0
1.5
arbidol (log μM)
IC50 = 3.5 μM
CC50 = 37 μM
Virus
cell viability

Fig S4

A. Drug Master Mix Plate

Drug Combo	Solvent (μL)	Drug A (μL)	Drug B (μL)	Drug C (μL)	Tube
**A→**	500	200	0	0	1
**B→**	500	0	200	0	2
**C→**	500	0	0	200	3
**A+B→**	300	200	200	0	4
**A+C→**	300	200	0	200	5
**B+C→**	300	0	200	200	6
**A+B+C→**	100	200	200	200	7
**Sol→**	700	0	0	0	8

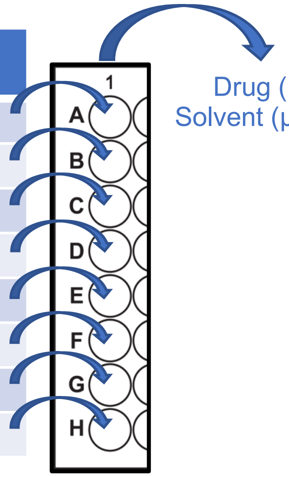

B. Drug Dilution Plate

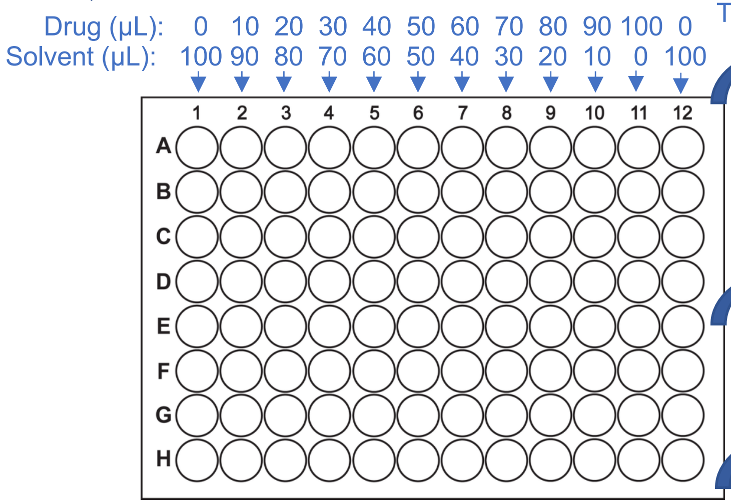

C. Final Plates

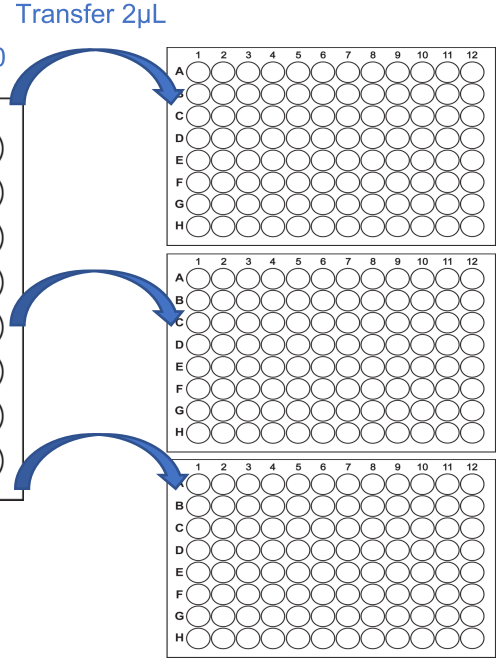

D. Final Plate Map

	1	2	3	4	5	6	7	8	9	10	11	12	Drug Combo
**A**	Mock	0.125X	0.25X	0.375X	0.5X	0.625X	0.75X	0.875X	1.0X	1.125X	1.25X	Sol	**←A**
**B**	Mock	0.125X	0.25X	0.375X	0.5X	0.625X	0.75X	0.875X	1.0X	1.125X	1.25X	Sol	**←B**
**C**	Mock	0.125X	0.25X	0.375X	0.5X	0.625X	0.75X	0.875X	1.0X	1.125X	1.25X	Sol	**←C**
**D**	Mock	0.125X	0.25X	0.375X	0.5X	0.625X	0.75X	0.875X	1.0X	1.125X	1.25X	Sol	**←A+B**
**E**	Mock	0.125X	0.25X	0.375X	0.5X	0.625X	0.75X	0.875X	1.0X	1.125X	1.25X	Sol	**←A+C**
**F**	Mock	0.125X	0.25X	0.375X	0.5X	0.625X	0.75X	0.875X	1.0X	1.125X	1.25X	Sol	**←B+C**
**G**	Mock	0.125X	0.25X	0.375X	0.5X	0.625X	0.75X	0.875X	1.0X	1.125X	1.25X	Sol	**←A+B+C**
**H**	Mock	Sol	Sol	Sol	Sol	Sol	Sol	Sol	Sol	Sol	Sol	Sol	**←Sol**

Fig S5

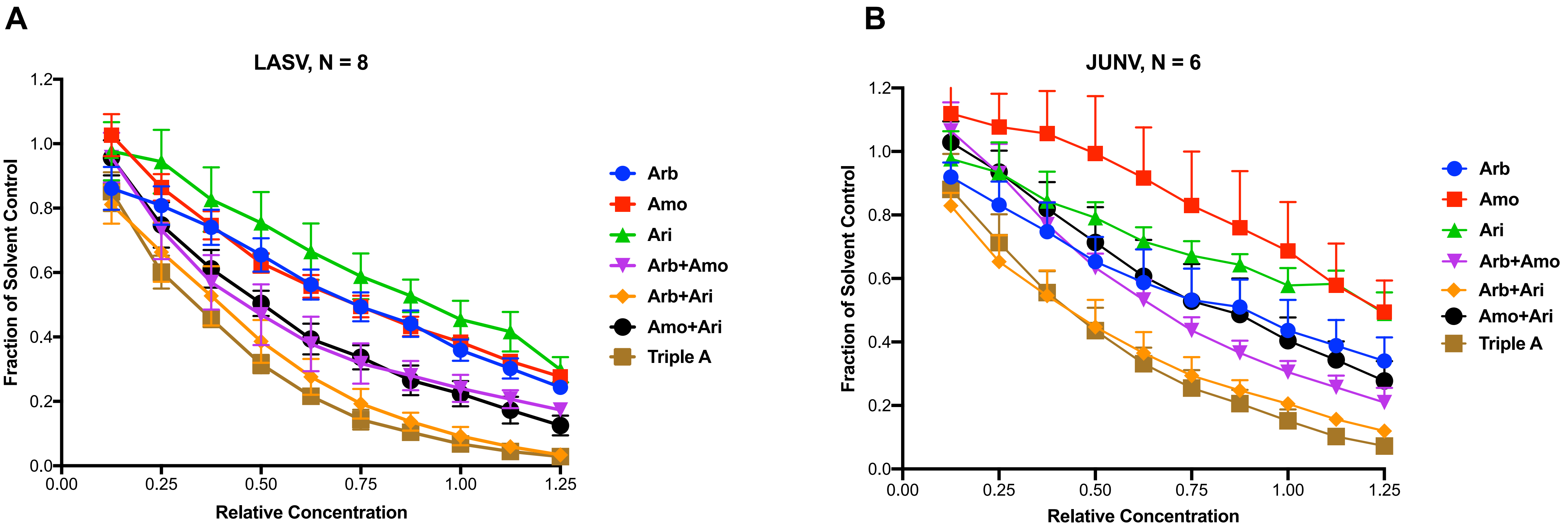
A
LASV, N = 8
1.2
1.0
0.8
0.6
0.4
0.2
0.0
Fraction of Solvent Control
0.00
0.25
0.50
0.75
1.00
1.25
Relative Concentration
Arb
Amo
Ari
Arb+Amo
Arb+Ari
Amo+Ari
Triple A
B
JUNV, N = 6
Fraction of Solvent Control
Relative Concentration
Arb
Amo
Ari
Arb+Amo
Arb+Ari
Amo+Ari
Triple A

Fig S6

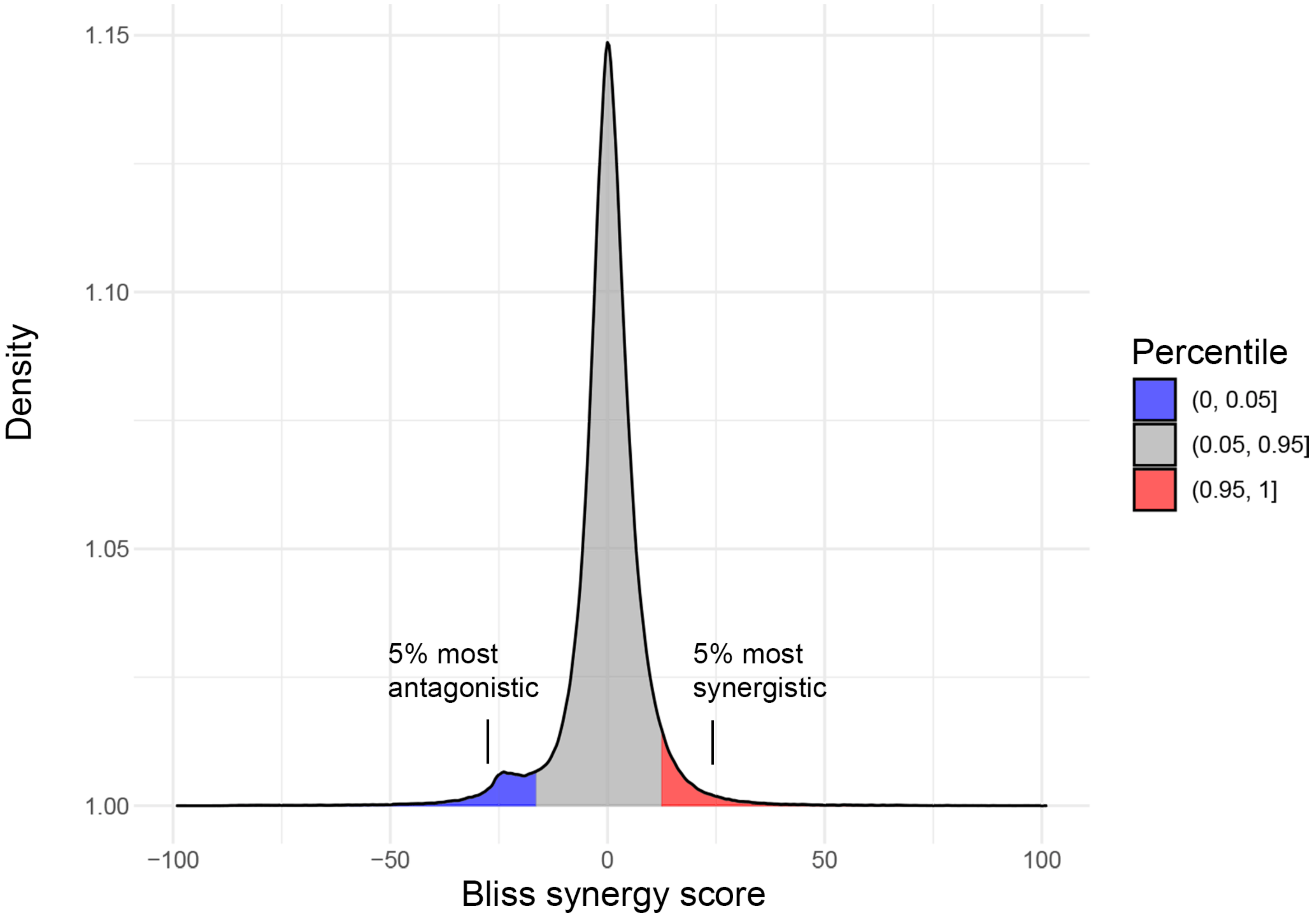

1.15
1.10
1.05
1.00
Density
5% most
antagonistic
5% most
synergistic
−100
−50
0
50
100
Bliss synergy score
Percentile
(0, 0.05]
(0.05, 0.95]
(0.95, 1]
